# A Novel Immunosuppressor, (5R)-5-Hydroxytriptolide, Alleviates Movement Disorder and Neuroinflammation in a 6-OHDA Hemiparkinsonian Rat Model

**DOI:** 10.14336/AD.2016.0929

**Published:** 2017-02-01

**Authors:** Ruijun Su, Min Sun, Wei Wang, Jianliang Zhang, Li Zhang, Junli Zhen, Yanjing Qian, Yan Zheng, Xiaomin Wang

**Affiliations:** ^1^Department of Physiology,; ^2^Department of Neurobiology, and; ^3^Key Laboratory for Neurodegenerative Disorders of the Ministry of Education, Capital Medical University, Beijing 100069, China.; ^4^Beijing Institute for Brain Disorders, Beijing100069, China.

**Keywords:** immunosuppressor, (5R)-5-hydroxytriptolide, neuroinflammation, Parkinson’s disease

## Abstract

Parkinson’s disease (PD) is one of the most common age-related neurodegenerative diseases. Promising therapies for PD still need to be explored. Immune dysfunction has been found to be involved in PD pathogenesis. Here, a novel immunosuppressor, (5R)-5-hydroxytriptolide (LLDT8), was used to treat 6-hydroxydopamine (6-OHDA)-induced hemiparkinson rats. We found that oral administration of LLDT8 significantly alleviated apomorphine-induced rotations at a dose of 125 µg/kg, and improved performance in cylinder and rotarod tests at a lower dose of 31.25 µg/kg, in 6-OHDA hemiparkinsonian rats. Moreover, loss of dopaminergic neurons in the substantia nigra pars compacta (SNpc) of the 6-OHDA rat was attenuated in response to LLDT8 treatment in a dose-dependent manner. In addition, inflammatory factors IL-1β, IL-6 and TNF-α, were significantly inhibited in LLDT8-treated hemiparkisonian rats, compared with vehicle. Notably, the level of dopamine (DA) in the striatum of PD rats was restored by LLDT8 treatment. Furthermore, we also detected that the disequilibrium of peripheral lymphocytes was reversed by LLDT8 administration. Taken together, the results imply that the immunosuppressor, LLDT8, can rescue dopaminergic neurodegeneration in 6-OHDA hemiparkinsonian rats, thus providing a potential therapeutic strategy for PD.

Parkinson’s disease (PD) is one of the most common neurodegenerative disorders, affecting approximately 1% of people worldwide over the age of 65 years [1]. It is characterized by a progressive loss of dopaminergic neurons in the substantia nigra pars compacta (SNpc), leading to dopamine (DA) depletion in the striatum, which ultimately causes classic motor symptoms including bradykinesia, postural instability, rigidity, gait disorder and resting tremors, as well as non-motor symptoms [2]. DA replacement strategies by oral administration of levodopa (the precursor of DA), DA receptor agonists, monoamine oxidase B (MAO-B) inhibitors and catechol *O*-methyltransferase (COMT) inhibitors are mainly palliative. Although these drugs can improve the clinical performance of individuals with PD, the therapeutic effect is disrupted several years later by the onset of motor fluctuations and dyskinesia, which result in alternating periods of reduced mobility and abnormal involuntary movements [3]. Unfortunately, until now there have been no promising drugs to cure, delay or stop the progress of the disease, because of poor understanding of PD pathogenesis.

Neuroinflammation, an enhanced innate immune response, is now accepted as a major event linked to chronic dopaminergic neurodegeneration in the progress of PD [4-6]. The discovery of T lymphocytes in the brain of postmortem human specimens and the mouse PD model further suggests a strong interaction between immune system destabilization and central nervous system (CNS) degeneration in PD pathogenesis [7, 8]. Although more studies are needed for better understanding of the mechanism linking these two paths, the modification of immune inflammation may be a potential therapeutic entry point in PD treatment. More recently, an immune suppressor, FK506, has been found to alleviate PD-like behavior and pathology [9], providing insight into the application of immunosuppressive drugs in PD treatment.

Triptolide is a major active component extracted from *Tripterygium wilfordii Hook F*, a traditional Chinese herbal medicine used for the treatment of inflammatory and autoimmune diseases for thousands of years [10]. Despite our finding that triptolide showed a neuroprotective effect in PD models [11], its successful application in the clinical treatment of individuals with PD is limited by toxicity. (5R)-5-hydroxytriptolide (LLDT8) is a new analog modified structurally from triptolide, and shows lower cytotoxicity and relatively higher immunosuppressive activity [12]. LLDT8 has been approved by the China Food and Drug Administration as an immunosuppressive drug for a clinical trial to treat rheumatoid arthritis. Given that triptolide had a significant neuroprotective effect on dopaminergic neurons in a lipopolysaccharide-mediated PD rat model [13], LLDT8 may also have a therapeutic effect in PD-like animals.

In this study, we investigated whether LLDT8 could alleviate PD-like behaviors and protect dopaminergic neurons against inflammation, mediated by 6-hydroxydopamine (6-OHDA), which is a neurotoxin and specific to dopaminergic neurons. Using a PD rat model, we injected 6-OHDA unilaterally into the neostriatal region, and detected that treatment with LLDT8 significantly improved behavioral deficits and prevented the loss of dopaminergic neurons in the SNpc in a dose-dependent manner. Moreover, the inflammatory response, reflected by an increase in proinflammatory factors and the activation of microglia and astrocytes in the striatum and SNpc, was obviously inhibited after LLDT8 administration for 5 weeks. Intriguingly, B lymphocyte enhancement in the peripheral blood of the PD rat model was detected and LLDT8 treatment restored the equilibrium of immune cells, implying that peripheral immune system regulation may be related to the mechanism of the therapeutic effect of LLDT8 in the model. Although a full understanding of the mechanism underlying immune system dysfunction and neurodegeneration in the brain is still required, our study provides insight into the role of immune system regulation in PD therapeutic strategy.

## MATERIALS AND METHODS

### Animals and drugs

**Figure 1.**
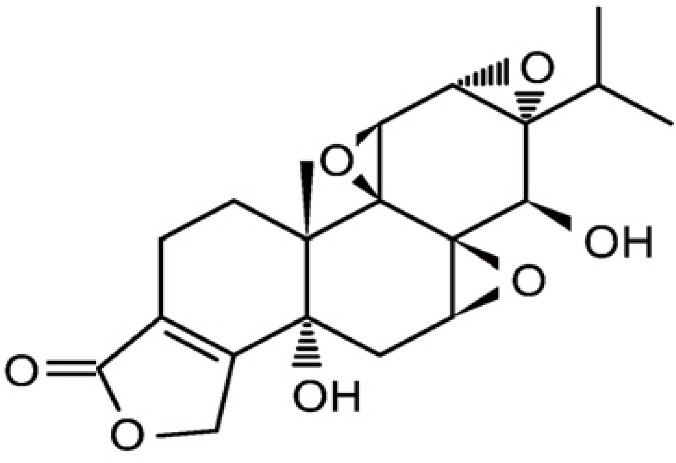
**Chemical structure of (5R)-5-hydroxytriptolide**. Formula: C_20_H_24_O_7_. Molecular weight: 376.39.

Eighty Male Sprague-Dawley rats (220-240g) were supplied from Beijing Vital River Lab Animal Technology Co., Ltd. (Beijing, China) and housed on a 12h:12h light/dark cycle with free access to food and water at 22~25 ºC, and habituated to the housing conditions for 3 days before the surgery. Every effort was made to minimize suffering and stress. All experimental procedures were approved by the Committee on Animal Care and Usage of Capital Medical University.

(5R)-5-hydroxytriptolide (LLDT8), which is composed of white amorphous powder, was kindly provided by professor Jian-ping Zuo (Shanghai Institute of Materia Medica, Chinese Academy of Sciences). LLDT8 is 99% pure by reverse phase high performance liquid chromatography (HPLC). The structure of LLDT8 is shown in Figure 1. The stock solution of LLDT8 (4 mg/ml) was dissolved in 2-Methyl-1.3-propanediol, stored at 4°C, and diluted to a desired concentration with 0.5% hydroxypropyl methyl cellulose (HPMC).

Rasagiline was purchased from TOCRIS. The stock solution of rasagiline (6 mg/ml) was dissolved in 0.5% HPMC, stored at -20°C, and diluted to a desired concentration with 0.5% HPMC.

### Surgical procedure

Rats were anesthetized with an intraperitoneal injection of 6% chloral hydrate (350 mg/kg), and then fixed in a stereotaxic apparatus (David Kopf Instruments, CA, USA) with flat skull position. The coordinates were as follows: anteroposterior (AP): 0.8 mm from the bregma; mediolateral (ML): 2.7 mm from the midline and dorsoventral (DV): -5.2, -4.5, respectively from the skull [14].

Thereafter, 6-OHDA (20 μg per rat in 4 μl saline with 0.01% (w/v) ascorbic acid in two sites, 2 μl/site) was infused into the left striatum with an infusion pump through a 10-μl Hamilton syringe at a constant flow rate of 0.2 μl/min. Sham-operated animals were submitted to the same procedure, and 4 μl vehicle (0.9% saline containing 0.01% (w/v) ascorbic acid) instead of 6-OHDA was infused into the striatum. The syringe was kept in the position for another 5 min per site and then was slowly retracted. During the experiment, temperature of animal body was maintained with a heating pad.

### The PD model determination and grouping

Apomorphine-induced rotation was used to appraise whether the PD model was successfully established. The numbers of rotation were recorded using a multichannel rotometer system (RotoMax, AccuScan Instruments, Inc,USA) [15]. Briefly, four weeks after surgery, all animals were injected subcutaneously with apomorphine hydrochloride (0.5 mg/kg, Sigma), and individually placed in the test cylinder. The drug-induced rotation was re-examined after 1 week. Rats that rotated in excess of 60 turns/30 min were considered the PD unilateral models. The established PD model rats were randomly divided into five groups (8-9 animals/group): (1) vehicle group treated with 0.5% HPMC, (2) drug treatment group treated with rasagiline (0.3mg/㎏), a MAO-B inhibitor, (3) three LLDT8 treated groups (31.25μg/㎏, 62.5μg/㎏, 125μg/㎏).

### The experimental procedures

**Figure 2.**
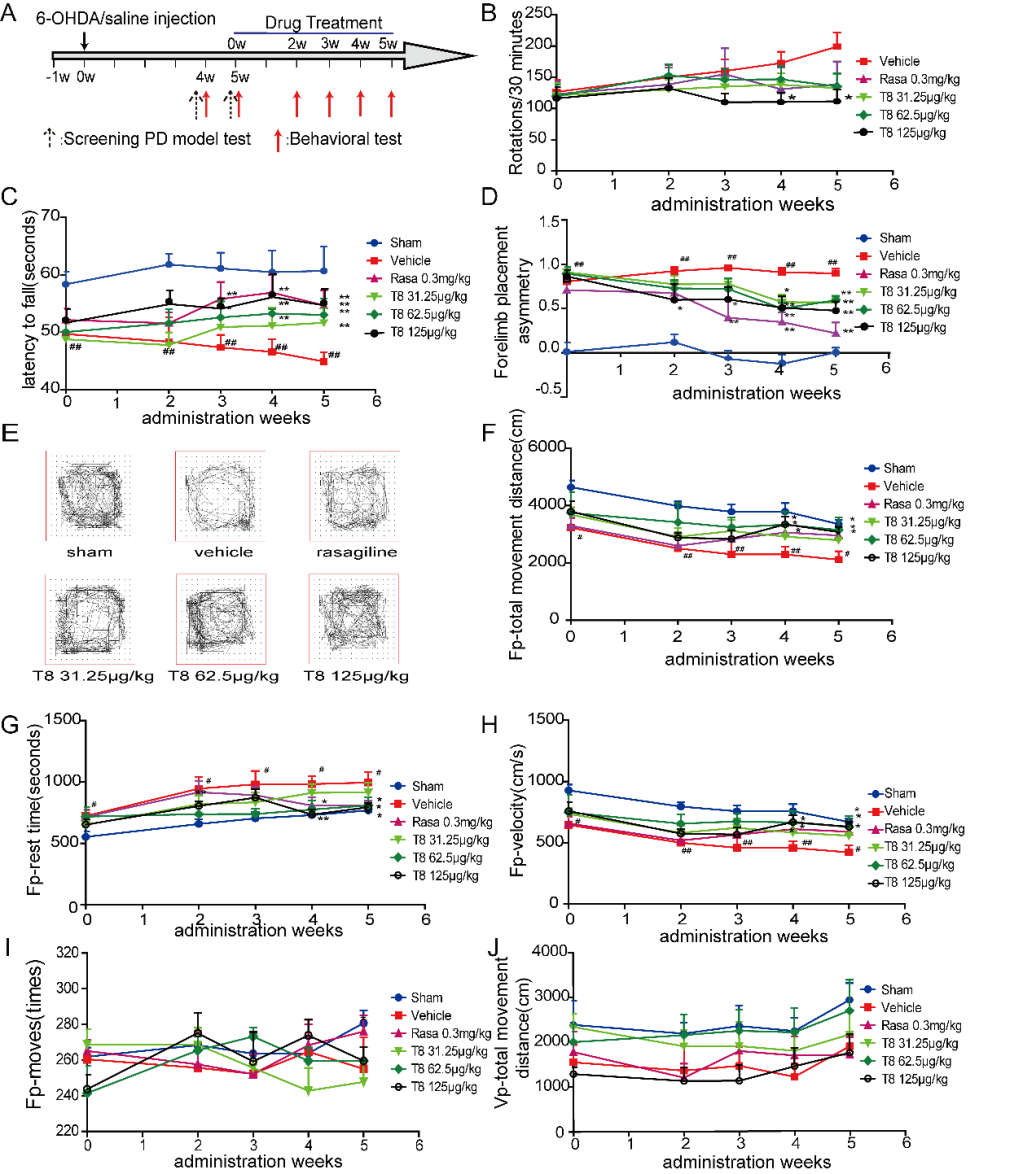
**LLDT8 administration enhanced movement performance in a 6-OHDA rat model. (A)** The schedule of experimental presentation. **(B-D)** The behavioral performance of the PD rats was determined by apomorphine-induced rotations **(B)**, rotarod **(C)** and cylinder test **(D)**. **(E)** Representative graph of the open field test. **(F-J)** Fp (Floor plane) -total movement distance (cm) **(F)**, Fp-rest time (s) **(G)**, Fp-velocity (cm/s) **(H)**, Fp-moves (times) **(I)** and Vp(Vertical plane)-total movement distance (cm) **(J)** were recorded in the open field test. Data are shown as mean ± SEM. ^#^*P* < 0.05, ^##^*P* < 0.01 vs. sham, ^*^P < 0.05, ^**^P < 0.01 vs. vehicle using one-way ANOVA with *post hoc* LSD *t*-test, or a Tamhane’s T2 test when variances were not equal, *n*=8-9.

All experimental animals were administrated orally once per day for 5 weeks. The behavioral tests of all groups were observed before drug treatment, then these tests were observed once weekly after two weeks of drug treatment, till these rats were sacrificed after 5 weeks of LLDT-8 or rasagiline treatment. The experimental procedure was shown as Figure 2A in detail.

### Behavioral tests

#### Apomorphine-induced rotations

Apomorphine-induced rotation was as described above. The drug-induced rotation was examined at 0, 2, 3, 4 and 5 weeks after treatment.

#### Rotarod test

Rotarod testing was conducted for the evaluation of motor coordination and balance [16]. Briefly, rats were trained to stay on the rotarod apparatus during a 5-min habituation trial (10 rpm) of 2 consecutive days before the first testing day. Rats were then subjected to a total of 4 rotarod test sessions with accelerating speeds (range, 4 - 40 rpm over a period of 2 min) at week 0, 2, 3, 4, and 5, respectively after treatment. The time duration of each animal staying on the rod was recorded as the latency to fall. Each test session was composed of 2 trials on the rotarod, a maximum duration of 2 min per trial, and a 20 min inter-trial interval. The best score achieved by each rat was used for further analysis.

#### Cylinder test

The spontaneous forelimb lateralization was measured by cylinder test, taking advantage of the natural exploratory instinct of rodents to a new environment [17]. Rats were placed individually in a glass cylinder (diameter 22 cm, height 26 cm) with two mirrors located at a 45° angle to allow a 360° vision behind cylinder. The session of initial forelimb touched to the walls of the cylinder (i.e., impaired right forelimb first, unimpaired left forelimb first, or simultaneous) was videotaped for 5 min. Each individual rearing episode was counted by a blinded researcher. The scores were calculated by following asymmetry ratio: (left-right)/(right+left+both). Scores on the forelimb asymmetry ratio range from -1 to 1. The positive ratio was consistent with greater use of the unimpaired forelimb over the impaired forelimb. In contrast, the negative asymmetry ratio suggests greater use of the impaired forelimb compared to the unimpaired forelimb. Thus, a high positive ratio would be consistent with a hemiparkinsonian lesion.

#### Open field test

Open field test was performed at 0, 2, 3, 4, and 5 weeks after treatment as described previously [13]. In brief, all of the behavioral procedures were performed between 9:00 AM and 3:00 PM, keeping room silence during the whole test. Locomotor activity was measured in automated activity chambers connected to an analyzer that transmitted the number of beam breaks (activity data) to a computer (VersaMon Version 2.01; Accuscan Instruments, OH, USA). The rats were placed individually in the center of the chamber. Locomotor activity was quantified as the number of beam interruptions (crossings) registered by a computer and recorded for 30min. The chambers were washed with 75% ethanol solution each time before next behavioral testing.

### Immunohistochemistry and imaging quantification

After 5 weeks’ administration, animals were deeply anesthetized with 6% chloral hydrate and perfused intracardially with warm 0.9% NaCl at room temperature, followed by 200 ml of cold 4 % paraformaldehyde (PFA/0.1M PBS). The brains were rapidly removed from the skull after decapitated and immersed in 4 % paraformaldehyde at least 24 h at 4 °C. Subsequently, the tissues were immersed with 20% and 30% sucrose sequentially, till sunk to the bottom of tube. Then OCT was used to embed the brain at -80°C over night. The coronal brain sections were made with a microtome at 30 μm thickness of the striatum and 50 μm thickness of the midbrain including SNpc. Immunohistochemistry (IHC) was performed on free floating sections, which were rinsed in 0.1 M phosphate buffer (PBS) three times for 5 min, followed by permeabilization with 0.3% Triton-X and washing with 0.1 M PBS three times for 5 min. Sections were treated with 3% hydrogen peroxide for 30 min and washed with 0.1 M PBS three times for 5 min. After blocked with 5% normal goat serum (Vector Laboratories) in 0.1 M PBS for 1h at room temperature, the sections were incubated with primary antibodies overnight at 4°C. The following primary antibodies were used in this study: anti-TH antibody (Mouse, 1:5000, T1299, SIGMA), anti-glial fibrillary acidic protein (GFAP) antibody (Mouse, 1:500, MAB360, Millipore), anti-CD11b (Mac1) antibody (Mouse,1:500, MCA275G, AbD Serotec). All antibodies were diluted in 5 mL 0.1 M PBS. Staining was done by the ABC KIT (Vector Laboratories, Inc.). The biotinylated anti-mouse IgG secondary antibodies (1:200) were used to recognize above primary antibodies, followed by washing 3 times and incubation with streptavidin-horseradish peroxidase complex (1:1000) at 37°C for 30 min. The immunoreactivities were visualized by 3, 3-diaminobenzidine (DAB) within 2 min. The sections were mounted and dehydrated in gradual concentration of ethanol.

The number of TH-positive cells in the SNpc was determined by stereological measurements using the Optical fractionator method in a computerized system (Stereo Investigator, Leica Microsystems CMS GmbH) as previously described [9, 18]. The sections were used for counting including the entire SNpc from the rostral tip of the pars compacta back to the caudal end of the pars reticulata. Every eighth section throughout the entire SNpc was counted, with a total of 6 sections for each animal. The estimates of the total number of neurons were calculated according to the optical fractionator formula and the coefficients of error < 0.10 were accepted. Both the injected and non-injected side of SNpc was quantified.

The area occupied by Mac1 or GFAP positive staining was defined by densitometry using Image-pro plus 6.0. All the analyses were performed by an investigator blind to different samples. Data were normalized to the contralateral normal side.

### Western blot analysis

Striatum were dissected and total protein lysates from all groups were harvested using RIPA lysis buffer (Beyotime). The protein was quantified with a Pierce™ BCA protein assay kit (Thermo scientific). Before loading, 60 μg of total protein and 5× loading buffer were mixed and heated at 95°C for 5 minutes. The mixtures were separated on a gel and transferred electronically to nitro cellulose membranes. The membranes were blocked with 5% non-fat milk for 1h at room temperature before incubation with anti-TH antibody (Mouse, 1:2000, Cat. No. T1299, SIGMA) overnight at 4°C. Then, the membranes were incubated with Goat anti mouse IgG 680 (Goat, 1:10000, Cat. No. 926-32220, LI-COR) conjugated secondary antibodies for 60 min at room temperature. The membrane was used to Odyssey (LI-COR, LINCOLN, NE USA) detection, and semi-quantitative analyses of densitometry were performed using Gel-Pro analyzer 4.

### DA wand its metabolite dihydroxyphenylacetic acid (DOPAC), homovanillic acid(HVA) measured by HPLC

The contents of DA and its metabolite DOPAC, HVA in the striatum were determined by HPLC apparatus (Model 5600A CoulArray Detector System ESA, MA, USA). Tissues were homogenized in 200 mM ice-cold perchloric acid then the homogenates were placed in an ice bath for 60 min. The samples were centrifuged at 15,000 × g for 20 min at 4°C, and the supernatant was transferred to another eppendorf tube. A solution (one-half volume of the supernatant) containing 20 mM potassium citrate, 300 mM potassium dihydrogen phosphate, and 2 mM ethylenediaminetetraacetic acid (EDTA)·2Na was added into the eppendorf tube and mixed thoroughly to precipitate the perchloric acid. After incubating 60 min in an ice bath, the mix was centrifuged at 15,000 × g for 20 min at 4°C. The supernatant was filtered through a 0.22-μm filter and injected into the HPLC system. The mobile phase was 125 mM sodium citrate buffer supplemented with 20% methanol, 0.1 mM EDTA·2Na, 0.5 mM 1-octanesulfonic acid sodium salt (Acros Organics, NJ, USA) and adjusted to pH 4.3. The flow rate was set at 1.2 ml/min.

### Detection of inflammatory cytokines(IL-6, IL-1β, TNF-α)by ELISA

The striatum of rats after 5 weeks of treatment were harvested. The tissues were homogenized in ice-cold normal saline by homogenizer for 3 min. Then the homogenate was repeated freezing and thawing twice, followed by centrifugation at 1,500 g for 15 min at 4°C. The supernatant was collected. The levels of IL-6,IL-1β and TNF-α were detected by enzyme-linked immunoabsorbent assay (ELISA) kits (Shanghai ExCell Biology Inc., Shanghai, China), respectively, according to the manufacturer’s instructions

### Detection of lymphocytes in blood by Flow cytometry staining

Blood samples were drawn into EDTA-K_2_ Vacutainer tubes (BD Biosciences). Afterwards, cells were incubated freshly for 20 min at room temperature with the following antibodies: RAT T/B/NK CELL CKTL (BD Pharmingen,558495) and RAT T LYM CKTL (BD Pharmingen, 558493). Red blood cells were lysed with Lysing Buffer (BD Pharmingen, 555899) for 10 min at room temperature. Quantification of blood cells was determined using BD Trucount tubes (BD Pharmingen,340334). Flow cytometric data were acquired using a BD Forutasa flow cytometer (BD Biosciences) and the BD CELL QUEST software, and analysed with the FlowJo software (Tree Star, Ashland, OR, USA).

### Statistical analysis

The data was analyzed with the software SPSS 21 and expressed as the mean ± S.E.M. Differences between mean values of normally distributed data were analyzed using one-way ANOVA with post hoc LSD *t*-test or when variances were not equal, a Tamhane’s T2 test was used. *P* < 0.05 was considered statistically significant.

## RESULTS

### LLDT8 alleviates movement disorder in PD rats

To evaluate the therapeutic effect of LLDT8 in the hemiparkinsonian rat model, we performed several behavioral tests, including APO-induced rotation, forelimb placement, motor co-ordination and locomotor activity (Fig. 2A). The number of APO-induced contralateral rotations in the LLDT8 treatment group (125 μg/kg) during a 30-minute testing period was significantly decreased compared with the vehicle group after administration, from week 4 (110.4 ± 14.4 turns/30 min) to week 5 (111.7 ± 19.1 turns/30 min) (Fig. 2B). Although rotations in the rasagiline treatment group also tended to decrease during week 5 of administration (138.7 ± 36.5 turns/30min), there was no significance when compared with the vehicle group.

Balance skills and motor co-ordination in the PD rats were appraised by rotarod test. The PD model rats treated with LLDT8 and positive control drug stayed on the accelerating rotarod for a significantly longer mean time than the vehicle group (*P* < 0.01; Fig. 2C) after 4 or 5 weeks of treatment. The latent period to falling from the rotarod in the high dosage LLDT8 group (58.9 ± 2.4 seconds) was significantly longer than that of the vehicle group (44.2 ± 2.7 seconds) after 3 weeks of administration.

The cylinder test was used to appraise the forelimb placement of the PD rats. The ratio of forelimb placement ([left-right]/[right+left+both]) of the LLDT8 treatment group and positive control group (rasagiline treatment group) was significantly lower than that of the vehicle group (*P* < 0.01; Fig. 2D) during the treatment period. In particular, the ratio of forelimb placement of PD rats treated with 125 μg/kg LLDT8 for 2 weeks was significantly lower than that of the vehicle group (0.60 ± 0.17 vs. 0.92 ± 0.04, *P* < 0.05; Fig. 2D).

Locomotor activity behavior was appraised using the open field test [19] with several parameters determined in the experiment. Total horizontal movement distance (cm) and velocity (cm/s) were significantly increased in the LLDT8 and rasagiline treatment groups compared with the vehicle group (*P* < 0.01; Fig. 2E, 2F & 2H). Horizontal rest time (s) was significantly decreased in the LLDT8 and rasagiline treatment groups compared with the vehicle group (*P* < 0.05; Fig. 2G). However, there was no difference in horizontal moves or vertical total movement distance (cm) between the drug treatment and vehicle groups (*P* > 0.05; Fig. 2I-J).

### TH^+^ neuron and fiber loss is prevented by LLDT8 administration

**Figure 3.**
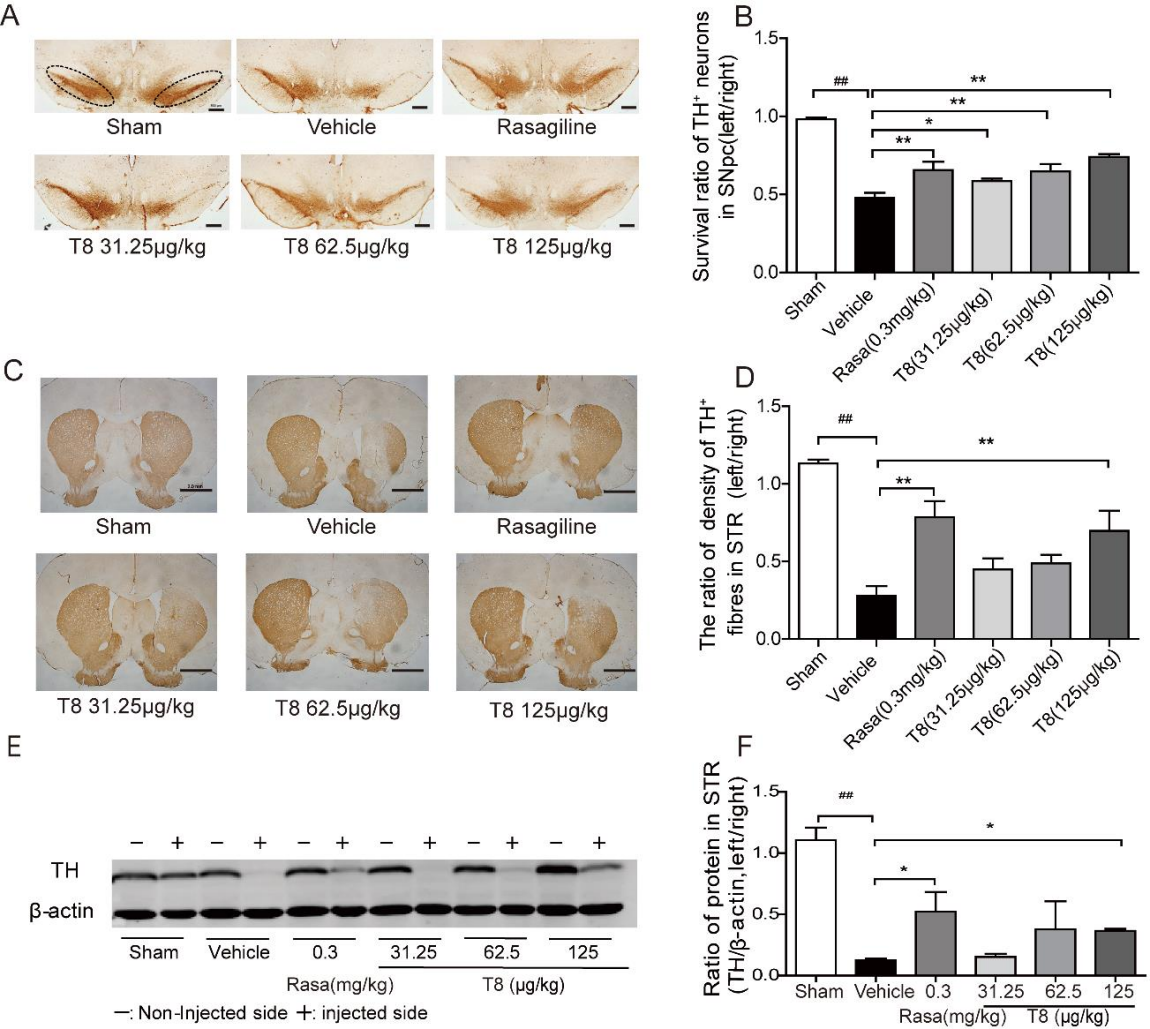
**PD-like pathology in the 6-OHDA rat model was attenuated by LLDT8 treatment. (A)** IHC staining for TH^+^ neurons in SNpc (as indicated by the dotted bracket). Scale bar = 500 μm. **(B)** The ratio (left/right) of stereological quantification of numbers of TH^+^ neurons in SNpc after systemic administration of different doses of LLDT8 (31.25, 62.5 and 125 μg/kg) or rasagiline, for 5 weeks. **(C)** IHC staining for TH^+^ fibers in striatum (STR) treated with LLDT8 for 5 weeks. Scale bar = 2.0 mm. **(D)** The ratio (left/right) of TH^+^ fiber density in STR after systemic administration of different doses of LLDT8 (31.25, 62.5, and 125 μg/kg) or rasagiline, for 5 weeks. **(E)** The scan picture of TH^+^ protein and **(F)** the statistical TH^+^ protein results of all experimental groups treated with LLDT8. Data are shown as mean ± SEM. ^#^*P* < 0.05, ^##^*P* < 0.01 vs. sham, ^*^P < 0.05, ^**^P < 0.01 vs. vehicle using one-way ANOVA with *post hoc* LSD *t*-test, *n*=4-6.

TH is the key enzyme in DA synthesis, and known as a marker for dopaminergic neurons. To confirm DA neuron survival, stereological quantification of TH^+^ neurons in SNpc was performed on all the groups. As anticipated, a significantly increased TH^+^ neuron survival in the SNpc of the LLDT8 and rasagiline treatment groups was evident compared with the vehicle group (*P* < 0.05, *P* < 0.01; Fig. 3A & 3B). Treatment with LLDT8 prevented dopaminergic neuron loss significantly, in a dose-dependent manner. Moreover, the survival ratio of TH^+^ neurons in the SNpc of the 125 μg/kg LLDT8 treatment group was 74%, compared with 48% in the vehicle group. TH^+^ fiber density in the striatum of the 125 μg/kg LLDT8 and rasagiline treatment groups was, as expected, higher than in the vehicle group (*P* < 0.01; Fig 3C & 3D). The TH^+^ protein level in the striatum, as measured using western blot, in the 125 μg/kg LLDT8 treatment group was increased significantly compared with the vehicle group (*P* < 0.05; Fig. 3E & 3F).

### LLDT8 treatment restores DA in the striatum

**Figure 4.**
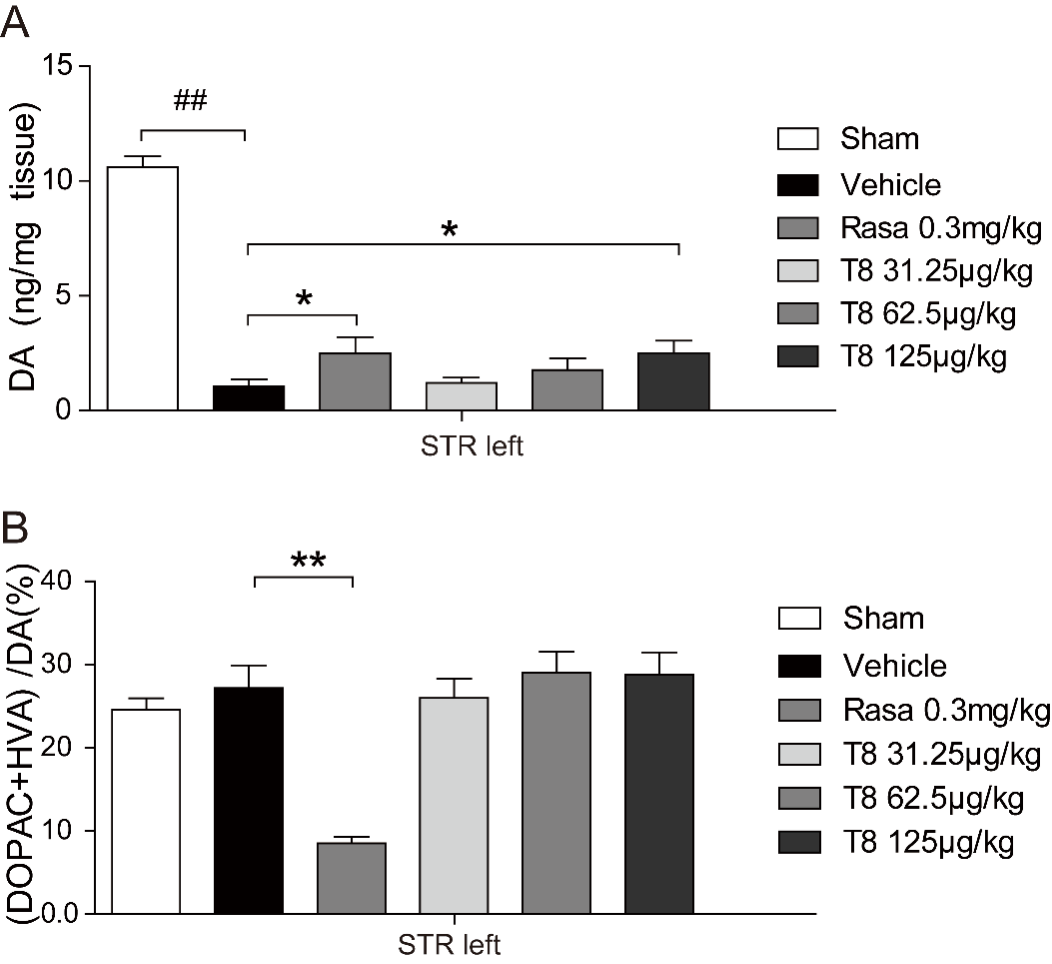
**The level of DA in striatum of the PD rat model was restored by LLDT8 treatment in a dose-dependent manner**. The model rats were treated by different doses of LLDT8 (31.25, 62.5, and 125 μg/kg) or Rasagiline (0.3mg/㎏) once daily for 5 weeks. **(A)** The concentrations of DA, and **(B)** its metabolite dihydroxyphenylacetic acid (DOPAC), homovanillic acid (HVA) in the striatum of all experimental rats were measured by HPLC, the metabolic rate of DA was calculated and used in statistical analysis. Data are shown as mean ± SEM. ^#^*P* < 0.05, ^##^*P* < 0.01 vs. sham, ^*^P < 0.05, ^**^P < 0.01 vs. vehicle using one-way ANOVA with *post hoc* LSD *t*-test, *n*=8-9.

It is well known that PD rat behavior is closely related to the amount of DA in the striatum. We used HPLC to measure DA concentration and found that, in the left striatum of the 125 μg/kg LLDT8 and rasagiline treatment groups, they were increased significantly compared with the vehicle group (Fig. 4A). Its Metabolic rates, calculated as (DOPAC + HAV)/DA, are shown in Fig. 4B. The metabolic rate in the left striatum of the rasagiline treatment group was significantly decreased (8%) compared with that of the vehicle group (27%), with no difference in the LLDT8 treatment group.

### LLDT8 attenuates neuroinflammation in the SNpc in the PD rat model

**Figure 5.**
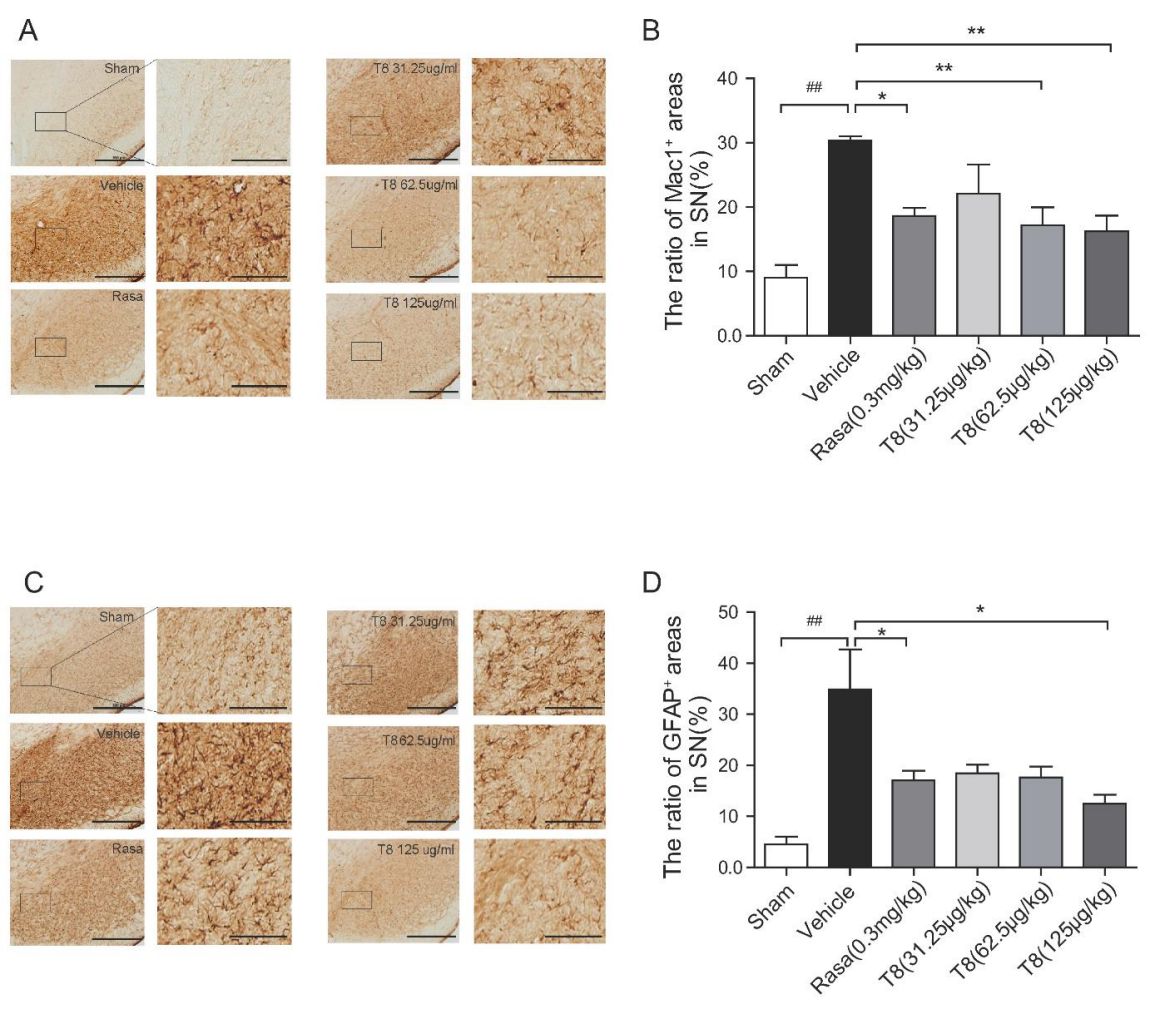
**The glial response in the PD rat model treated with LLDT8**. The representative IHC staining for Mac1^+^ microglia **(A)** and GFAP^+^ astrocytes **(C)** in SNpc. Scale bar = 500 μm. The quantitative percentage of area occupied by Mac1^+^ cells **(B)** and GFAP^+^ cells **(D)** in the indicated region of SNpc. Data are shown as mean ± SEM. ^#^*P* < 0.05, ^##^*P* < 0.01 vs. sham, ^*^P < 0.05, ^**^P < 0.01 vs. vehicle using one-way ANOVA with *post hoc* LSD *t*-test, *n*=4-6.

Astrocyte and microglia populations in the rat brain were observed and presented as the percentage of area occupied by GFAP^+^ (specific for astrocytes) and Mac1^+^ (specific for microglia) cells in ipsilateral SNpc. Morphological alterations of glia cells, activated by 6-OHDA, were observed, consistent with a report that glial reactivity is a crucial event in the inducement process in the 6-OHDA PD model [20]. The area occupied by Mac1^+^ cells was significantly lower in the SNpc of the LLDT8 (62.5 μg/kg and 125 μg/kg) and rasagiline treatment groups compared with that of the vehicle group (*P* < 0.01, Fig. 5A & 5B). Meanwhile, the area occupied by GFAP^+^ cells (*P* < 0.05, Fig. 5C & 5D) in the SNpc of LLDT8 (125μg/kg)-treated or rasagiline-treated PD rats was lower compared to the vehicle rats.

### LLDT8 inhibits generation of proinflammatory cytokines in the ipsilateral striatum of a PD rat model

**Figure 6.**
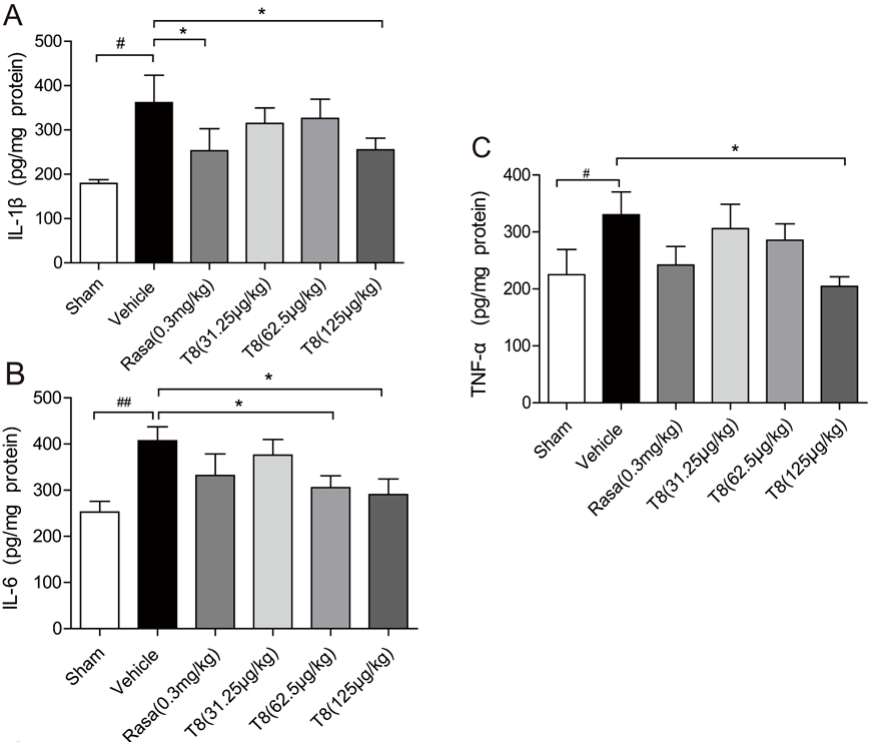
**Dose-dependent effects of LLDT8 on inflammation factors in the PD rat model**. The concentrations of **(A)** IL-1β, **(B)** IL-6 and **(C)** TNF-α in ipsilateral striatum of all experimental animals. Data are shown as mean ± SEM. ^#^*P* < 0.05, ^##^*P* < 0.01 vs. sham, ^*^P < 0.05, ^**^P < 0.01 vs. vehicle using one-way ANOVA with *post hoc* LSD *t*-test, *n*=8-9.

Mounting evidence shows that inflammation has a key role in the PD process [21]. Once activated, microglia express several pro-inflammatory mediators, such as IL-1β, IL-6 and TNF-α, which are thought to be closely associated with inflammation-related diseases [22-24]. We used ELISA to detect the inflammation factors IL-1β, IL-6 and TNF-α in the striatum, and LLDT8 (125μg/kg)-treated model rats had significantly lower levels than model rats without treatment, for all three factors. However, rasagiline-treated model rats showed a lower level only for IL-1β (Fig. 6A-C).

### LLDT8 inhibits an increase in the lymphocyte population of PD rats

**Figure 7.**
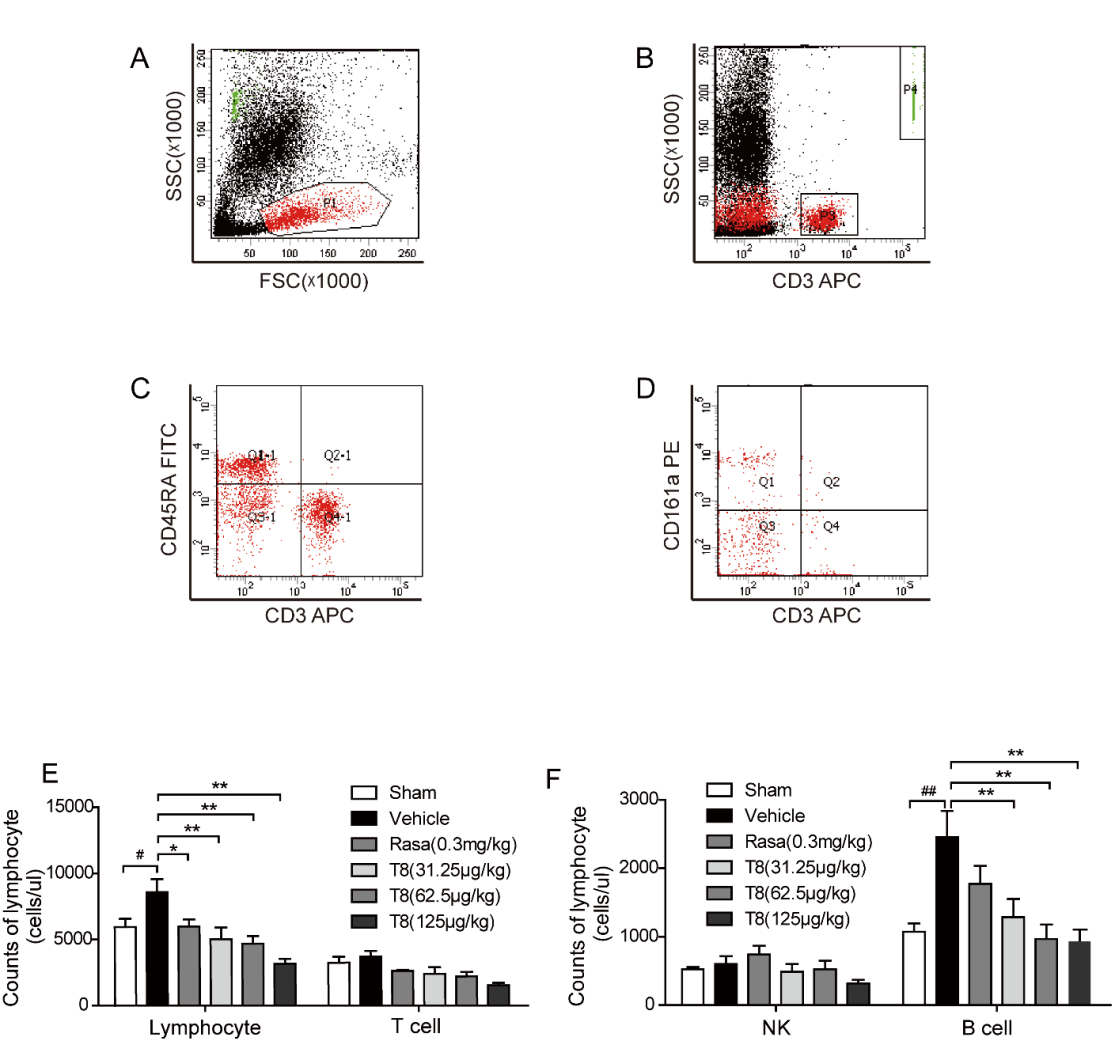
**Treatment of LLDT8 balanced the peripheral immune cells of the PD rat model. (A-D)** Gating strategy is represented for each cell type assessed using flow cytometry. Representative flow cytometry plots showing the lymphocytes subsets in PD rat blood are shown. P1 is represented for total lymphocytes **(A)**; P3: T lymphocytes **(B)**; Q1-1: B cells **(C)**; Q1: natural killer (NK) cells **(D)**. **(E)** The counts of total lymphocytes and T cells, **(F)** the counts of NK and B cells were statistically analyzed. Data are shown as mean ± SEM. ^#^*P* < 0.05, ^##^*P* < 0.01 vs. sham; ^*^P < 0.05, ^**^P < 0.01 vs. vehicle using one-way ANOVA with *post hoc* LSD *t*-test, *n*=8-9.

We investigated the lymphocyte subpopulation absolute count using flow cytometry (Fig. A-D). In the vehicle group, there was a significant increase in the number of lymphocytes compared with the sham group. In contrast, LLDT8 inhibited an increase in total lymphocytes (*P* < 0.01, Fig. 7E) in the treatment group. In addition, the number of B cells in the vehicle group was, significantly, more than twice that of the sham group. LLDT8 inhibited an increase in the B cell population in a dose-dependent manner (*P* < 0.01, Fig. 7F); however, this does not happen in rasagiline-treated group.

## DISCUSSION

The symptoms of PD are caused by loss of dopaminergic neurons in the SNpc, leading to decreased DA levels in the neostriatum [21]. From the perspective of DA replacement therapy, the course of PD cannot be modified, but only slowed down in the early stages [25]. Considering the important role played by the immune response in PD progression, the targeting of an event occurring in immune-CNS interaction [26] in the PD process should provide a novel and efficient pathway. We demonstrated in this study, for the first time as far as known, the therapeutic effect of the immunosuppressor LLDT8 in a hemiparkinsonian rat model. PD-like behavior and nigrostriatal degeneration were alleviated, accompanied by suppression of the inflammatory response.

The 6-OHDA hemilesioned PD rat model has been widely used for decades to investigate mechanisms and therapeutic strategies, as it mimics PD-like motor deficits relatively reliably and can partly recapitulate the progression of PD pathology [27, 28]. In this study, several PD-like behaviors were comprehensively evaluated using the model, including the following: apomorphine-induced rotational behavior, which is related to the degree of nigrostriatal DA loss and is considered the "gold standard" in the hemilesioned rat model[28], the rotarod test, which is often used to appraise the balance skills and motor co-ordination in the Parkinsonism rat [16]; the cylinder test, which can be used to determine the forelimb placement asymmetry of PD rats [17]; and the open field test, which can reflect alterations in DA function. It is well known that the decreased DA concentration probably causes locomotion hypoactivity [19]. The behavioral performance of the LLDT8-treated model rats was improved (Fig. 2), even more so than in the rasagiline-treated model rats (Fig. 2), suggesting that LLDT8 at a dose of 125 μg/kg has more obvious therapeutic effects than rasagiline, although a comparable protective action on TH^+^ neurons and fibers in nigrostriatal system was exhibited (Fig. 3).

Basically, PD-related symptoms are determined by DA levels in the striatum, which is the terminal field of SNpc dopaminergic projection [19]. Therefore, we detected striatal DA in this study, and as expected, the results showed that the improved behavior resulted precisely from restoration of DA in the striatum (Fig. 4A). However, the DA catabolism process, reflected by the percentage of (DOPAC + HVA)/DA in the striatum of the PD rats, was accelerated by rasagiline but not by LLDT-8 administration (Fig. 4B), indicating that the mechanism of the dopaminergic neuroprotective effect of LLDT-8 in the PD model is very different from that of the MAO-B inhibitor, rasagiline. Given the crucial role of inflammatory response in the PD pathogenesis process [29-33] and the properties of the immunosuppressor, we sought to investigate whether anti-inflammatory action is the main mechanism of LLDT-8 in the 6-OHDA-lesioned nigrostriatal system. Consistent with our proposal, LLDT-8 significantly suppressed both morphological glial activation and the release of all proinflammatory cytokines that we detected. However, these results were not completely reproduced in rasagiline-treated model rats, despite obvious glial morphological alteration and IL-1β inhibition by the MAO-B inhibitor (Fig. 5 & 6). The limited anti-inflammatory effect together with the dopaminergic neuroprotective action of rasagiline in the PD rat model may be secondary to its anti-oxidative activity [34].

Neuroinflammatory processes play a key role in PD pathogenesis. Mounting evidence from PD-like animal models, human postmortems and therapeutic studies support the presence of a neuroinflammatory cascade in PD [29, 30]. Microglia are generally known as brain macrophages that play an important role in the immune system of the CNS, providing defense against pathogen invasion and promoting destructive inflammation [31,32]. Glial responses have been considered a double-edged sword in neurodegenerative disease. Although the activation of glial cells may benefit dopaminergic neurons in the early stages of PD, continued glial activation contributes to a vicious circle of neurodegeneration and inflammation [20, 30, 33]. In this study, astrocyte and microglia activation was observed in the SNpc of the vehicle group, accompanied by proinflammatory cytokine enhancement in the dopaminergic terminal field of the striatum, while LLDT-8 attenuated these phenotypes comprehensively. This result implies a disruption to the devastating cycle, by LLDT-8 treatment. The anti-inflammatory effect of LLDT-8 on the nigrostriatal system should not be the only path to protect dopaminergic neurons, as LLDT-8 has been defined as an immunosuppressor. Our finding of stabilization of peripheral lymphocytes by LLDT-8 suggests that immune system homeostasis may be disrupted in the PD process and an immunoregulatory strategy could restore CNS function. However, several questions need to be elucidated, such as how intrastriatal injection of 6-OHDA leads to the dysequilibrium of the peripheral immune system, whether immune homeostasis imbalance is involved in early-stage PD progression, and, if this is the case, whether peripheral immune factors or a certain type of lymphocyte could be used as biomarkers for PD diagnosis at a very early stage.

In conclusions, we demonstrated that an immunosuppressor, LLDT-8, alleviated PD-like behaviors and dopaminergic neurodegeneration, as well as neuroinflammation, in the nigrostriatal system. Intriguingly, the equilibrium of the peripheral immune system in the hemilesioned PD rat model was restored by LLDT-8 treatment. The study not only provides a novel therapeutic application for LLDT8 in the treatment of PD, but may provide a therapeutic entry point focusing on immune regulation, in early-stage PD.
